# EQ-5D-5L population norms and health inequalities for Trinidad and Tobago

**DOI:** 10.1371/journal.pone.0214283

**Published:** 2019-04-29

**Authors:** Henry Bailey, Mathieu F. Janssen, Althea La Foucade, Paul Kind

**Affiliations:** 1 Arthur Lok Jack Global School of Business, The University of the West Indies, Mt. Hope, Trinidad and Tobago; 2 HEU, Centre for Health Economics, The University of the West Indies, St. Augustine, Trinidad and Tobago; 3 Section Medical Psychology and Psychotherapy, Department of Psychiatry, Erasmus MC, Rotterdam, The Netherlands; 4 Department of Economics, The University of the West Indies, St. Augustine, Trinidad and Tobago; 5 Academic Unit of Health Economics, Institute of Health Sciences, University of Leeds, Leeds, United Kingdom; Sciensano, BELGIUM

## Abstract

The EQ-5D instrument is now used in many health systems as a health outcomes measure. Recently an EQ-5D valuation study was conducted for Trinidad and Tobago, but thus far there have been no population norms published for Trinidad and Tobago or for any Caribbean country. The objective of this study is to provide a set of population norms, and to investigate inequalities in health in Trinidad and Tobago. The EQ-5D-5L questionnaire was included in the 2012/2013 Adult Population Survey of the Global Entrepreneurship Monitor for Trinidad and Tobago. This survey covered a representative sample of 2,036 adults aged 18 and over. Demographic data and self-reported health using EQ-5D-5L were collected. The Trinidad and Tobago value set was used to obtain EQ-5D index values. The Kakwani index and logistic regression models were used to evaluate inequalities in health. Mean EQ-5D index values and EQ-VAS values were calculated by age group, ethnicity, gender, income, educational attainment, employment status and place of residence. The 10 most commonly observed EQ-5D-5L states accounted for 90% of the respondents. The mean VAS value for the sample was 83.6 and the mean EQ-5D-5L index value was 0.95. Pain/discomfort was found to be the EQ-5D dimension with the highest prevalence of reported problems with 22% of the population reporting pain at any level. Self-care was the dimension with the lowest prevalence of problems reported at any level (3%). Health declines with increasing age, and men reported fewer problems and higher levels of self-reported health than women. Age, gender and education level were found to be important drivers of health status as measured by the EQ-5D instrument. Being in a very low income group was also observed to affect EQ-VAS values among younger respondents. The population norms provided in this study can be used by clinicians, academics and policy makers in several ways. They can be used in comparing different demographic groups or patient groups, or as a basis for tracking the progress of patients through a treatment regimen. They can also provide a baseline for cost utility analysis of health interventions for Trinidad and Tobago.

## Introduction

The EQ-5D instrument (EuroQol Group) is a preference-based measure that is now used in healthcare decision making in many countries. This instrument is required, encouraged or recommended for use in cost utility analysis by health technology assessment agencies in countries as diverse as Egypt [1], Chile [2], Thailand [3], the Baltic states [4], England & Wales [5], The Netherlands [6] and others.

The EQ-5D instrument captures a description of the state of a respondent’s health along 5 dimensions (which facilitates comparison over time or with other respondents, or groups) and the respondent’s valuation of their state of health. Having the EQ-5D states and self-reported health ratings for a population provides clinical practitioners and policy makers with useful tools. These values are referred to as population norms and they can be used by clinical professionals to determine whether an individual patient scores better or worse than the average for their demographic group. Clinicians can also use EQ-5D data to track a patient’s progress over the course of an illness or during treatment [7]. Policy makers can use EQ-5D population norm data as an indicator of the state of health of different groups as inputs for resource allocation and other policy type decisions. Clinicians, policy makers and administrators can use EQ-5D to monitor performance [8]. Thus far EQ-5D population norms have been developed for at least 24 countries [9] but not for any countries in the Caribbean. Population norm studies using EQ-5D have found differences in EQ-5D outcomes among different socioeconomic groups [9].

This is the first study to investigate health inequality in Trinidad and Tobago using a generic health outcomes instrument. Some studies have investigated inequalities relating to specific illnesses in Trinidad and Tobago. For example, one study [10] found links between poverty, gender and HIV incidence, with unemployment being a key driver of HIV incidence among women in Trinidad and Tobago. Another study [11] looked at self-reporting rates for diabetes, hypertension and cardiac illness in Trinidad and Tobago. Disease prevalence was found to be higher among Indo-Trinidadians, and among those with the lowest education levels. Hypertension was found to be significantly more prevalent among women.

The aim of this study is to present the EQ-5D population norms for Trinidad and Tobago and to provide insight into inequality in health within the population of Trinidad and Tobago.

## Methods

In 2012 the EQ-5D 5 level (EQ-5D-5L) instrument was included in the Adult Population Survey of the Global Entrepreneurship Monitor (GEM) study for Trinidad and Tobago. This survey covered a representative sample of 2,036 adults. This study included among other things, views and perceptions of the general public concerning various aspects of entrepreneurship. Details of the GEM study are provided elsewhere [12]. Data were collected in a national face-to-face survey. The sample comprised persons aged 18 and over from all administrative areas in the country based on the population of the area and the breakdown by gender and age group according to the most recent census data. Enumeration districts were selected from Central Statistical Office of Trinidad and Tobago Enumeration District maps based on population and demographics to ensure that the sample matched the national population. Interviewers visited one in every four households up to the quota for each selected enumeration district and the person over age 18 with the most recent birthday in the household was interviewed. Call back cards were left in the households when the person with the most recent birthday was not at home. Demographic data, answers on each EQ-5D dimension and EQ-VAS score were collected for each respondent. The demographic variables collected from each respondent were age, gender, health insurance status, employment status, town, ethnicity, income, education level and household size. Health insurance status was captured in the GEM survey using response categories: yes through employment, yes through family member, yes through having a personal or family policy, don’t know and refused. For analysis in this study, these were collapsed into yes, no and missing. Employment status was captured in the GEM survey using response categories for self-employed (full time), employed by others (full time), self-employed (part time), employed by others (part time), seeking employment, retired or disabled, student, full-time home-maker, refused. For analysis in this study, these were collapsed into working, unemployed, student, and retired. Ethnicity was captured in the GEM survey using response categories for Afro-Trinidian, Indo-Trinidadian, Caucasian, mixed and other. For this analysis these were collapsed into Indo-Trinidadian, Afro-Trinidadian and other. Income was captured in the GEM survey using 6 income ranges (all in Trinidad and Tobago dollars per year): under $24000; $24,001-$60,000; $60,001-$120,000; $120,001-$180,000; $180,000-$240,000; over $240,000; and refused. These 6 groups were used in the analysis. Education level was captured in the GEM survey using 10 response categories. These are: none; incomplete primary; complete primary; incomplete secondary; complete secondary; vocational; post-secondary or technical; university undergraduate; university post-graduate; and refused. For analysis, these were collapsed into less than complete secondary, complete secondary or vocational/technical and tertiary. Household size was captured in the GEM survey with a numerical response. For analysis, these responses were grouped into 1–2 persons, 3–4 persons and 5 or more. For all of the variables, the response categories: don’t know, missing and refused were treated in the analysis as missing.

There were no exclusions (i.e. no respondent data were removed from the dataset, however, some respondents did not answer questions on some variables). Where possible, the demographics of the sample were compared with national data available from the Central Statistical Office of Trinidad and Tobago. This includes age groups, gender, ethnicity, education level and geography by Regional Health Authority (RHA).

The EQ-5D instrument comprises 5 dimensions coded in the following order: mobility, self-care, usual activities, pain or discomfort, and anxiety or depression. Two versions of the instrument exist. The EQ-5D-3L is the original version with 3 answer categories on each dimension. The 3 level version of the EQ-5D instrument allows 3^5^ = 243 states. An EQ-5D valuation study was conducted recently which produced a value set giving the preferences of Trinidad and Tobago citizens among the 243 states [13]. The recently developed EQ-5D-5L has 5 answer categories on each dimension. These 5 answer categories are: no problems (level 1), slight, moderate, severe and extreme (level 5) problems on each dimension.

The 5 dimensions and 5 levels produce 5^5^ = 3,125 possible combinations that each describe a state of health. A respondent’s EQ-5D state is given by the respondent’s level on each dimension in the order: mobility, self-care, usual activities, pain/discomfort, anxiety/depression. Thus if a respondent is in state 12543, this would mean:

1: No problems in walking about
2: Slight problems with bathing or dressing oneself
5: Unable to perform usual activities
4: Severe pain or discomfort
3: Moderately anxious or depressed

In EQ-5D-5L state 11111 there are no problems on any of the 5 dimensions. This is considered to be the ‘full health’ state. All other states have at least level 2 (slight problems) on at least one dimension. Therefore, each EQ-5D state other than state 11111 will represent some decrement from full health. The value set gives the value that society places on each state. These values can be used to perform cost utility analysis in the economic evaluation of health programs and interventions. This would bring the preferences of Trinidad and Tobago citizens into economic evaluation and resource allocation decisions for Trinidad and Tobago.

The Trinidad and Tobago value set [13] was used to derive the EQ-5D index values. This value set was developed using EQ-5D-3L but in the GEM Study EQ-5D-5L questionnaire was used. In order to use the Trinidad and Tobago values with the 5-level states, the value set had to be transformed via a crosswalk algorithm to map them on to 5-level values [14]. The values in the 3-level and 5-level crosswalk value sets are compared in Table 1.

**Table 1:** A comparison of values in the EQ-5D-3L value set and the EQ-5D-5L crosswalk value set for Trinidad and Tobago.

	3-Level	5-Level
**Mean**	0.475	0.524
**Median**	0.497	0.565
**Range:**		
**Maximum after 11111**	0.896	0.917
**Minimum**	-0.163	-0.163

The EQ-5D instrument also includes a Visual Analogue Scale (VAS) on which a respondent can indicate their self-rated health at the time using a 0–100 (worst-to-best imaginable health) scale. The EQ-5D instrument has proven to be sensitive in many different conditions and disease areas. In some conditions the evidence is mixed (e.g. certain mental disorders, visual disorders and COPD [15]). To improve sensitivity in such applications, there has been some research into adding “bolt-on” dimensions to the existing EQ-5D [16,17].

Mean values for EQ-VAS and EQ-5D index values were calculated for different demographic groups (by age, gender, geographic region, education level etc). Differences in mean EQ-VAS and EQ-5D index values among demographic groups were tested for statistical significance using ANOVA and t-tests, and a difference in the mean EQ-VAS or EQ-5D index value between two groups was considered to be statistically significant using conventional significance levels (p-values of 0.05 or less). These investigations were carried out for age and gender sub-groups to capture any effect that would remain after adjusting for age and gender which are known to be important drivers of self-reported health.

Inequalities in health were investigated using two approaches. In the first approach, EQ-VAS values were used to determine the extent of differences in the level of self-reported health between demographic groups. This was done using the Kakwani Index [18]. To do this the following regression model was run:

$$\frac{2\sigma_{R}^{2}}{\bar{EQ\;VAS}}{EQ\;VAS}_{i}=\alpha_{i}+\gamma_{k}R_{i}+\varepsilon_{i}$$

where $\bar{EQ\;VAS}$ is the mean EQ-VAS value, R_i_ is the relative fractional rank of the ith individual (ranked by the individual’s EQ-VAS score), σ^2^ is the variance of the fractional rank variable (*EQ VAS)*, α is the constant of the regression model, ε is the error term and γ_k_ is the concentration index (with k referring to underlying explanatory components). This can then be decomposed to give inequalities by demographic groups. The interpretation of this index is similar to that of the Gini ratio of inequality on a scale of 0 to 1 [19]. A higher value indicates greater inequality.

In the second approach to investigating inequalities, logistic regression was used to obtain odds ratios for reporting problems on the five dimensions of the EQ-5D instrument for different demographic groups. To perform the logistic regression analyses, the 5 dimensions of the EQ-5D instrument were dichotomized into two levels (no problems and ‘any’ problems) and these were used as dependent variables. The explanatory variables included in this model were gender, age group, education level and income group. The reference group for these analyses was male in the youngest age group, highest education and highest income group. The explanatory variables were dichotomized into being in the reference group versus not being in the reference group.

Ordinary Least Squares (OLS) regression was used to develop a model which would give an approximation of the expected VAS value based on age and gender.

All statistical analyses were conducted using STATA 14.

## Results

A representative sample of 2,036 respondents completed the survey; 2,242 respondents were approached, 164 refused (7%) and 42 gave incomplete interviews. A comparison of the sample with the national population is presented in Table 2.

**Table 2:** Sample and population characteristics and EQ-5D population norms for Trinidad and Tobago, 2012.

	Sample		Population	EQ-5D Index			EQ-VAS			Ceiling
	N	%	%	Mean	S.E.	p Value	Mean	S.E.	p Value	Effect
**Overall**	2036	100%	N/A	0.950	0.002		83.6	0.350		71.6%
**Age Group**						0.000			0.000	
18–24	320	16%	16%	0.977	0.003		87.7	0.745		84.7%
25–34	466	23%	25%	0.978	0.003		85.3	0.685		83.9%
35–44	364	18%	21%	0.965	0.004		84.9	0.750		77.2%
45–54	369	18%	16%	0.941	0.005		83.7	0.807		66.9%
55–64	266	13%	11%	0.925	0.006		80.2	1.024		59.0%
>64	248	12%	11%	0.884	0.008		76.7	1.199		44.4%
**Gender**						0.004			0.000	
Male	1002	49%	51%	0.961	0.002		84.6	0.478		76.2%
Female	1034	51%	49%	0.940	0.003		82.6	0.509		67.2%
**Health Insurance**						0.046			0.000	
Yes	458	23%	N/A	0.967	0.003		85.3	0.644		78.8%
No	1556	77%	N/A	0.945	0.002		83.1	0.414		69.3%
**Employment**						0.000			0.000	
Working	1273	72%	N/A	0.968	0.002		85.5	0.497		79.3%
Unemployed	110	6%	N/A	0.965	0.006		86.6	1.191		73.6%
Student	67	4%	N/A	0.979	0.007		85.6	1.770		86.6%
Retired	308	18%	N/A	0.878	0.007		76.4	1.070		41.6%
**Region (Regional Health Authority)**						0.006			0.350	
Eastern RHA	110	5%	8%	0.950	0.009		82.8	1.473		72.7%
North Central RHA	535	26%	23%	0.946	0.004		83.6	0.622		68.2%
Northwest RHA	449	22%	24%	0.956	0.004		81.4	0.773		71.9%
Southwest RHA	843	41%	41%	0.949	0.003		85.0	0.553		73.3%
Tobago RHA	96	5%	4%	0.959	0.008		82.7	1.810		72.9%
**Ethnicity**						0.524			0.000	
Afro	921	45%	39%	0.959	0.003		84.0	0.941		74.4%
Indo	850	42%	39%	0.939	0.003		83.1	0.500		68.0%
Other	262	13%	22%	0.958	0.005		83.8	0.570		74.0%
**Income**						0.009			0.000	
<24k	216	20%	N/A	0.927	0.007		80.6	1.163		62.5%
24k-60k	493	46%	N/A	0.949	0.004		83.2	0.711		70.8%
60k-120k	487	46%	N/A	0.953	0.004		84.9	0.665		74.9%
120k-180k	249	23%	N/A	0.964	0.005		85.5	0.934		79.1%
180k-240k	96	9%	N/A	0.972	0.006		84.2	1.460		81.3%
>240k	68	6%	N/A	0.962	0.010		85.3	1.761		76.5%
**Education**						0.041			0.000	
Less than Complete Secondary	625	31%	38%	0.877	0.012		78.9	1.990		41.5%
Complete Secondary or Vocational	1032	51%	51%	0.913	0.006		80.5	0.983		56.7%
Tertiary	376	18%	11%	0.952	0.006		82.9	1.256		67.7%
**Household Size**						0.027			0.000	
1–2	469	23%	N/A	0.933	0.005		81.9	0.784		64.8%
3–4	876	43%	N/A	0.957	0.003		84.4	0.501		74.3%
5 or more	688	34%	N/A	0.953	0.003		83.7	0.613		73.0%

The EQ-5D index is the mean of the values taken from the cross-walk value set applied to each EQ-5D state reported by the respondents.

The EQ-5D VAS is the average VAS value for each subgroup.

In Table 2, the column headed Ceiling Effect gives the percentage of respondents in each sub group who reported their health state as 11111 (no problems on all five EQ-5D dimensions). No respondent reported being in state 55555, so there was no ‘floor effect’.

The mean VAS value obtained in this study was 83.6 and the mean EQ-5D index value was 0.950.

Differences in mean VAS and index values for the variables in Table 2 were investigated using ANOVA and t-tests. All of the variables in Table 2 failed Bartlett’s test of equality of variance for the subgroups, so Welch’s ANOVA or t-tests (which allow for inequality of variances) were used to test whether the differences between group means were statistically significant. The p-values in Table 2 give the significance of the F statistic in Welch’s ANOVA. Thus with a p-value of <0.001 for VAS scores, the differences in VAS scores among age groups in Table 2 were highly significant. This was also observed for EQ-5D index values. Similarly, at group level, the differences in mean VAS values and EQ-5D index values were both found to be significantly different at the 5% level for gender, health insurance and employment status, income and education groups, and household size. For region, the differences in VAS scores (but not EQ-5D index vales) were significant at the 5% level and for ethnicity, the differences in EQ-5D index values (but not VAS scores) were significant at the 5% level.

When these mean values were calculated for age and gender groups, many of the apparent differences in Table 2 were no longer observed. While the data in Table 2 suggest that having health insurance is associated with higher index and VAS values, this effect is only significant to the 5% level for males in the 45–54 age group, females in the 55–64 age group (VAS); and for males in the 35–44 age group, all respondents in the 45–54 age group and females in the >64 age group (index value). Differences in VAS between the highest and lowest income groups were significant to the 5% level after adjusting for age and gender only for males in the 18–24 and 25–34 age groups and for females in the 18–24 age group. For education however, differences in VAS and index scores (between the highest and lowest education level groups) were generally observed to be significant at 5% and 10% levels for both genders but only in higher age groups (p-values of 0.1 or less for the 45–54 age group and p-values of less than 0.05 for the 55–64 age group).The differences in VAS and EQ-5D index values for age and gender subgroups by income group and education level and insurance status are presented as Tables A, B and C in S1 Appendix respectively.

The remaining demographic factors in Table 2 (Regional Health Authority, ethnicity, employment status and household size) did not produce groups with mean VAS or index values that differed significantly at the 5% level when these differences were calculated for age and gender sub groups. Differences in VAS and EQ-5D index values for these factors in Table 2 appear to be driven by age and gender distribution.

The 5-level EQ-5D instrument allows 5^5^ = 3,125 possible states. Of these, 120 were observed in the sample. 72% of the respondents reported being in state 11111 (with no problems on all 5 dimensions). 10 states accounted for 90% of the sample. These are displayed in Table 3.

**Table 3:** The 10 states making up 90% of the sample.

State	Frequency	Cumulative %	EQ-5D Index Value	EQ-VAS Value
11111	1,457	72	1.000	87.0
11121	140	78	0.874	80.3
11112	67	82	0.917	81.6
21121	41	84	0.822	75.9
11131	26	85	0.843	70.0
11122	23	86	0.850	85.3
21221	20	87	0.785	70.0
11113	13	88	0.896	82.5
31131	13	88	0.798	61.9
21111	12	89	0.796	87.5

### Age and gender

The rates of self-reporting problems at all levels of the 5 EQ-5D dimensions by age group and gender are presented in Table 4. Half of the respondents over age 64 and 75% of the women in this age group reported having pain/discomfort at some level.

**Table 4:** The percentage of respondents reporting problems on each dimension by gender and age group.

	Age Group														
	18–24		25–34		35–44		45–54		55–64		>64		Total		
	Male	Female	Male	Female	Male	Female	Male	Female	Male	Female	Male	Female	Male	Female	Total
**Mobility**															
**1**	99%	97%	98%	98%	97%	94%	91%	86%	84%	74%	79%	54%	93%	86%	89%
**2**	1%	3%	1%	1%	2%	3%	6%	10%	11%	14%	12%	29%	5%	9%	7%
**3**	0%	0%	0%	0%	0%	3%	1%	3%	4%	9%	7%	12%	1%	4%	3%
**4**	0%	0%	0%	0%	1%	0%	2%	2%	1%	4%	2%	4%	1%	1%	1%
**5**	0%	0%	0%	0%	0%	0%	0%	0%	0%	0%	0%	1%	0%	0%	0%
**Levels 2 to 5**	1%	3%	2%	2%	3%	6%	9%	14%	15%	26%	21%	46%	7%	14%	11%
**Self Care**															
**1**	99%	98%	100%	100%	100%	99%	97%	97%	99%	95%	93%	89%	98%	97%	97%
**2**	1%	1%	0%	0%	0%	1%	2%	3%	0%	5%	5%	8%	1%	3%	2%
**3**	0%	1%	0%	0%	0%	0%	1%	0%	1%	1%	2%	2%	0%	0%	0%
**4**	0%	0%	0%	0%	0%	0%	0%	0%	0%	0%	0%	1%	0%	0%	0%
**5**	0%	0%	0%	0%	0%	0%	0%	0%	0%	0%	1%	1%	0%	0%	0%
**Levels 2 to 5**	1%	2%	0%	0%	0%	1%	3%	3%	1%	5%	7%	11%	2%	3%	3%
**Usual Activities**															
**1**	98%	96%	98%	98%	97%	96%	92%	89%	88%	84%	85%	76%	94%	91%	93%
**2**	1%	4%	2%	2%	3%	2%	5%	8%	7%	12%	10%	16%	4%	7%	5%
**3**	1%	0%	0%	0%	0%	1%	2%	3%	4%	2%	2%	3%	1%	1%	1%
**4**	0%	0%	0%	0%	0%	1%	1%	1%	0%	1%	2%	5%	0%	1%	1%
**5**	0%	0%	0%	0%	0%	0%	0%	0%	0%	0%	1%	0%	0%	0%	0%
**Levels 2 to 5**	2%	4%	2%	2%	3%	4%	8%	11%	11%	16%	15%	24%	6%	9%	7%
**Pain/Discomfort**															
**1**	91%	90%	91%	98%	84%	82%	82%	66%	72%	60%	59%	42%	82%	74%	78%
**2**	6%	8%	8%	8%	12%	11%	10%	24%	18%	26%	30%	30%	12%	16%	15%
**3**	3%	1%	0%	1%	3%	5%	5%	6%	9%	9%	8%	21%	4%	6%	5%
**4**	0%	1%	0%	1%	1%	2%	1%	4%	1%	6%	2%	6%	1%	3%	2%
**5**	0%	0%	0%	0%	0%	0%	1%	0%	0%	0%	1%	0%	0%	0%	0%
**Levels 2 to 5**	9%	10%	9%	10%	16%	18%	18%	34%	28%	40%	41%	58%	18%	26%	22%
**Anxiety/Depression**															
**1**	94%	88%	92%	89%	93%	89%	90%	88%	92%	85%	87%	83%	92%	88%	90%
**2**	5%	9%	6%	6%	6%	7%	7%	8%	5%	11%	11%	12%	6%	9%	8%
**3**	1%	1%	1%	3%	1%	2%	2%	2%	1%	2%	2%	4%	1%	2%	2%
**4**	0%	1%	1%	1%	0%	2%	1%	2%	1%	1%	0%	1%	0%	1%	1%
**5**	0%	1%	0%	0%	1%	0%	2%	0%	0%	1%	0%	0%	0%	0%	0%
**Levels 2 to 5**	6%	12%	8%	10%	7%	11%	10%	12%	7%	15%	13%	17%	8%	12%	11%

Women were twice as likely as men to report problems with mobility (14% vs 7%, p = 0.000) and also more likely to report problems on all other dimensions. For both genders, the dimension with the lowest frequency of reported problems was self-care.

The dimension with the highest number of reported problems (levels 2 through 5) was pain/discomfort (22%). Mobility and anxiety/depression were both 11% and problems with usual activities and self-care had much lower frequency of reported problems at 7% and 3% respectively.

Table 5 shows the VAS scores and index values by age group and gender. This shows that the decrease in health as people age is more rapid for women than men in Trinidad and Tobago, and that this decrease accelerates after the mid-fifties age group.

**Table 5:** VAS scores and EQ-5D index values by age group and gender.

Age Group	Gender	Mean	Std. Dev.	95% Confidence Interval
**VAS Scores**				
18–24	Male	88.0	13.5	85.9–90.1
25–34	Male	86.3	14.4	84.4–88.1
35–44	Male	84.9	14.4	82.7–87.0
45–54	Male	85.3	14.6	83.2–87.5
55–64	Male	80.7	15.8	78.0–83.4
>64	Male	79.2	17.6	75.9–82.6
18–24	Female	87.5	13.2	85.4–89.5
25–34	Female	84.3	15.1	82.4–86.3
35–44	Female	84.9	14.3	82.8–87.0
45–54	Female	82.2	16.2	79.9–84.5
55–64	Female	79.6	17.6	76.6–82.7
>64	Female	74.7	19.6	71.4–77.9
				
**EQ-5D Index Values**				
18–24	Male	0.981	0.050	0.973–0.989
25–34	Male	0.980	0.051	0.973–0.986
35–44	Male	0.970	0.064	0.961–0.980
45–54	Male	0.950	0.094	0.936–0.964
55–64	Male	0.943	0.088	0.928–0.958
>64	Male	0.913	0.110	0.892–0.934
18–24	Female	0.973	0.065	0.963–0.983
25–34	Female	0.977	0.059	0.969–0.984
35–44	Female	0.961	0.073	0.950–0.971
45–54	Female	0.931	0.094	0.918–0.945
55–64	Female	0.905	0.107	0.887–0.924
>64	Female	0.862	0.134	0.839–0.884

### Inequality

The Kakwani Index for Trinidad and Tobago was estimated to be 0.103. Decomposition analysis of the Kakwani Index for Trinidad and Tobago showed that of the health inequality observed in Trinidad and Tobago, 5.25% is ‘explained’ by demographic factors included in this study. This was decomposed into age (4.7%), income (0.3%), gender (0.2%) and education (less than 0.1%). 15.6% of the inequality in the EQ-VAS based Kakwani index is ‘explained’ by the EQ-5D dimensions.

Results of the logistic regression analyses are presented in Table 6.

**Table 6:** Odds ratios for different demographic groups reporting any problems on each EQ-5D dimension.

	Mobility		Self Care		Usual Activities		Pain/Discomfort		Anxiety/Depression	
	O.R.	95% C.I.	O.R.	95% C.I.	O.R.	95% C.I.	O.R.	95% C.I.	O.R.	95% C.I.
Age	7.12**	2.61–19.46	1.90	0.58–6.23	2.77**	1.27–6.03	2.95**	1.83–4.77	1.83*	0.99–3.37
Gender	1.71**	1.23–2.38	1.73*	0.91–3.31	1.57**	1.08–2.28	1.44**	1.13–1.85	1.60**	1.13–2.27
Education	3.94**	2.20–7.06	11.91**	1.63–87.03	2.48**	1.40–4.39	2.27**	1.61–3.18	1.61**	1.02–1.24
Income Group	2.71	0.65–11.40	1.00		4.29	0.58–31.46	1.15	0.57–2.32	0.02**	0.00–0.15

O.R.: Odds Ratio

C.I.: Confidence Interval

*significant at p<0.1

**significant at <0.05

The reference category for this logistic regression comprised the youngest age group, male, highest education group, and the two highest income groups combined. The two highest income groups were combined for the analysis in Table 6 because of the small numbers of respondents in these groups. The independent variables in Table 6 were dichotomized into being in the reference group or not being in the reference group. An odds ratio in Table 6 gives the change in odds of reporting a problem on the EQ-5D dimension associated with not being in the reference group.

### VAS model

Regression analysis was used to create a simple formula that can be used to obtain the expected VAS value for an individual based on age and gender. The formula obtained is:

ExpectedVAS=95−[0.2×Age−(1.7×Female)]

For an investigator or clinician who wants an estimate of the average VAS score for a 60-year-old female, this would be: 95−[0.2×60]−1.7 = 81.3. For a male of age 45, the Female coefficient would drop out so the expected VAS score would be: 95−[0.2×45] = 86. The purpose of this model is to give users a rough guide or indication of an average VAS value that can be expected given the demographic characteristics. This model did not produce normally distributed residuals or pass tests for linearity.

## Discussion

The mean VAS value of 83.6 is high when compared with the values of EQ-VAS ratings for 21 countries [9, 20, 21, 22] which ranged from 71.1 (in Hungary) to 83.7 (in Denmark). Similarly, the EQ-5D index value 0f 0.950 is high when compared to the overall values in other studies which ranged from 0.758 (in Portugal) to 0.958 (in Korea). People’s preferences and values among different states of health are known to be driven by many factors including factors relating to national culture [23]. Also, comparison of overall VAS and EQ-5D index values should be made with caution since not all population norm studies are based on representative samples, and the overall mean values would be driven in part by demographic differences such as age distribution.

A respondent who reports being at level 1 on all 5 EQ-5D dimensions would have an EQ-5D index value of 1.000. However, such a respondent may have an EQ-VAS value of less than 100 because EQ-VAS and the EQ-5D index value measure different things. The EQ-VAS captures the subjective value that the respondent places on his or her own health. The EQ-5D index value captures the societal value that the population (in this case, the Trinidad and Tobago population) will place on a hypothetical state.

Population norm studies usually show EQ-VAS values and EQ-5D index values declining with age. This pattern was also observed in the Trinidad and Tobago study. In Table 2, the EQ-5D index value for age group 25–34 is 0.001 higher than that for the 18–24 age group, but this difference is smaller than the standard error of the EQ-5D index value for both age groups and is therefore neither clinically nor statistically relevant.

The mean VAS value reported by men was higher than that reported by women. The difference was small (2 VAS points) but statistically significant (p = 0.002). Similarly, the mean EQ-5D index value for the health states reported by men was slightly (but statistically significantly) higher than that for the states reported by women (0.961 vs 0.940, p<0.001). Men were more likely to report their health state as 11111 (76.2% vs 67.2%). The pattern of men having slightly higher self-reported health than women has been observed in many countries [9]. The poorer self-rated health of women has been demonstrated to be associated with musculoskeletal and other pain disorders in some countries [24]. However, there are some countries in which women report higher mean EQ-VAS scores than men (e.g. Slovenia, New Zealand and Thailand [25,26,27]).

The Health Insurance variable was included in the survey to test the hypothesis that having health insurance would lead to higher health status in Trinidad and Tobago. Health services are perceived to be of higher quality [28], faster delivery [29] and better customer service [30] in the private sector (where fees are paid for services) than in public institutions (where services are provided with no out of pocket charges) in Trinidad and Tobago. An individual with health insurance can visit a private clinic, and obtain laboratory tests, imaging services, pharmaceuticals etc. and pay only the copayment (which may be as low as 10–20% of the cost of treatment). A person with no health insurance coverage may have the choice of paying the full cost of healthcare in the private sector or seeking care in a public institution. Intervention thresholds are likely to be lower for those who are covered by health insurance (usually through employment or being a dependent of a covered employee). Indeed, respondents covered by health insurance reported higher levels of health by VAS and EQ-5D index however this effect was found to be driven by age and gender distribution within the insured and un-insured groups as no systematic differences in VAS or EQ-5D Index values were observed when t-tests were carried out at the age-gender sub group level.

The effect of education level on self-reported health was measured by the differences VAS and EQ-5D index values reported by the highest and lowest education groups in this study. This relationship is statistically significant at the 5% and 10% levels in older age groups but not in the very oldest age group. The impact of education level on health status has been summarized elsewhere [11, 31] to include such factors as self-management, compliance with treatment and lifestyle/health-related behaviors. The absence of a significant difference in the 18–24 age group may be because there are not many people within this age group who would have graduated from university. The increasing strength of this relationship at higher age groups would also be consistent with the idea that lifestyle choices made in adolescence and early adulthood may affect health status in later years. If higher education leads to better lifestyle choices and these choices result in improved health later on, then the pattern of a divergence in health status between education groups would be expected. However, the relationship does not extend to the very highest age group in this study. Part of the reason for this may be due to the fact that this is an open-ended age group which would have included very senior respondents who in turn would have health conditions that come with age. The relationship would be further complicated for this age group if it comprised a disproportionately higher ratio of better educated people (assuming that the benefits of the earlier lifestyle choices include longevity). Further study would shed light on the drivers of health status in this age group for Trinidad and Tobago.

Income has been found to have direct and indirect effects on health [30]. When calculated by age group and gender the differences in mean VAS scores between the highest and lowest income groups were significant at the 5% and 10% levels for respondents in the youngest age group. However, no similar pattern was seen for the EQ-5D index values. Low income males in the 35–44 age group had a higher mean VAS score than their high income counterparts, and this difference was significant at the 10% level. Further investigation is warranted in the relationship between income and VAS and EQ-5D index values in Trinidad and Tobago.

In Table 6 the odds ratios for age groups are significant at the 5% and 10% levels for all of the EQ-5D dimensions except for self-care. The odds ratios for education are all also significant at the 5% and 10% levels. Gender (i.e. being female) was observed to have highly significant odds ratios for all EQ-5D dimension except for self-care. This is the first study of inequality in health using EQ-5D to include income. The odds ratios for income groups were found to be significant at the 5% level only for mobility and usual activities.

The Kakwani Index of 0.103 places Trinidad and Tobago in the lower quartile when compared with results of a study of 17 countries for which similar analyses have been undertaken [9]. In that study, the median Kakwani Index was 0.112 and the range was 0.090 (Korea) to 0.173 (Spain). Considering the range of the Kakwani index scale between 0 and 1, with 0 indicating complete equality in health, inequality in Trinidad and Tobago is relatively low. Other indices of inequality also place Trinidad and Tobago close to—or less than—the median when compared internationally. Thus from the UNDP statistics for 2015 [32] Trinidad and Tobago is in the second quartile of the index of Human Inequality and in the third quartile for the Gini Coefficient and the index of Inequality of Life Expectancy.

Decomposition analysis of the Kakwani Index for Trinidad and Tobago showed that only 5.25% of the health inequality as measured by EQ-5D Index values in Trinidad and Tobago is associated with the demographic factors included in this study. The rest of the inequality in health would be due to other (non-demographic) factors. The contribution of income to overall health inequality is greater than that of education, even though at the level of the age-gender subgroups in Tables A and B in S1 Appendix, the effect of income was seen in fewer age-gender subgroups than the effect of education.

Decomposition analysis of the health inequality captured in the EQ-VAS-based Kakwani index for Trinidad and Tobago showed that 15.6% of the inequality is ‘explained’ by the EQ-5D dimensions. This figure is low compared to a study of 17 countries for which similar analyses have been undertaken [9]. In that study the median percentage was 35% and the range was 14.6% (in Thailand) to 54.3% (in Greece and Slovenia). The 15.6% for Trinidad and Tobago was decomposed into pain/discomfort (7.9%), mobility (4.4%), usual activities (2.1%), Anxiety/depression (0.9%), and self-care (0.3%).

This study had some limitations. One limitation is the absence of marital status (which is known to affect health status). Future studies of this kind in the Caribbean region should include marital status and may also benefit from including experience with chronic illness. Another possible limitation may lie in the use of a crosswalk value set for the index values. Finally, it should be noted that significant differences found between groups in this study do not necessarily reflect a minimal (clinical) important difference.

## Conclusion

The aim of this study was to provide a set of EQ-5D population norms for Trinidad and Tobago. These utility scores and VAS values are also provided by demographic groups. This study provides clinicians and policy makers with data that can support clinical and resource allocation decision making. It also provides a basis for comparison for future studies that look at the health impact of specific illnesses. The data for Trinidad and Tobago follow similar patterns to those seen in many other countries (declining with age, higher for men, lower for lower income and education groups). Analysis of the health inequality in Trinidad and Tobago as captured by EQ-5D was found to be mainly driven by income and education (out of the demographic factors included in this study) and by pain/discomfort and mobility (as dimensions of health).

Further attention/investigation may be warranted for the issues of pain/discomfort and mobility especially among women in the over 64 age group. This may also hold for the burden of disease among the most disadvantaged in Trinidad and Tobago society: the lowest educational attainment and lowest income groups. Further investigation may also be warranted for the impact of education level on the health of the highest age group.

## Supporting information

S1 Appendix Table A. Absolute differences in VAS and EQ-5D Index Values between the highest and lowest income groups by age and gender. Table B. Absolute differences in VAS and EQ-5D Index Values between the highest and lowest education groups by age and gender. Table C. Absolute differences in VAS and EQ-5D Index Values between respondents with- and without- private health insurance by age and gender.
(DOCX)
