# Supplementary material for: EQ-5D-5L population norms and health inequalities for Trinidad and Tobago
Source: PLoS One. 2019 Apr 29;14(4):e0214283. doi: 10.1371/journal.pone.0214283 (PMC6488064; doi:10.1371/journal.pone.0214283)
Supplement: S1 Appendix — Table A. Absolute differences in VAS and EQ-5D Index Values between the highest and lowest income groups by age and gender. Table B. Absolute differences in VAS and EQ-5D Index Values between the highest and lowest education groups by age and gender. Table C. Absolute differences in VAS and EQ-5D Index Values between respondents with- and without- private health insurance by age and gender. (DOCX) [file pone.0214283.s001.docx]

**S1 Table A.**

**S1 Table B.**

**S1 Table C.**
